# From plaques to pocks: carrying over bacteriophage assay techniques to the study of influenza and other animal viruses

**DOI:** 10.1017/mdh.2025.10024

**Published:** 2026-01

**Authors:** Neeraja Sankaran

**Affiliations:** School of Arts and Sciences, Ahmedabad University, Central Campus Navrangpura, Ahmedabad, Gujarat, India

**Keywords:** Bacteriophages, Plaque-counting assays, Felix d’Herelle, Macfarlane Burnet, Influenza virus, Pock counting assays

## Abstract

This article assesses the impact of the discovery of bacteriophages, which emerged from an investigation into a 1915 outbreak of bacillary dysentery in France, on influenza virus research. Specifically, it details the way in which the phages became a vehicle for importing certain assay techniques into the study of influenza and other viruses that cause infectious diseases in humans and other animals, thereby enabling the scaling up of vaccine production for these diseases. Very soon after his 1917 report of the discovery of bacteriophages, Felix d’Herelle developed an assay technique based on their ability to form countable plaques on solid media when incubated along with the dysentery bacteria. This basic technique was further refined by Macfarlane Burnet in the late 1920s. Still later, in the wake of a 1935 influenza outbreak in Australia, Burnet applied the principles of serial dilution and plaque counting, honed during his work on the phages, to develop a technique for cultivating influenza viruses in fertilised eggs and assaying them by counting the pocks induced on the chick embryo membranes. The ability to grow and assay these viruses proved crucial in developing the first successful vaccines against influenza. In the 1950s, bacteriophage assay techniques were once more carried over to the assaying of viruses on cultured cells by Renato Dulbecco and Marguerite Vogt. The importance of quantification in science, as well as the ability to apply the results of investigations in one area of biology to another, relatively unrelated field, is also discussed.

## Introduction

The role of bacteriophages, viruses that infect bacteria rather than the cells of plants or animals (and called phages for short), as the right ‘tool for the job’^1^ in the origins of molecular biology has dominated the history of biology for so long now that it is easy to forget the phages were ever anything else. The foundational text that made this claim and popularised this idea will soon be celebrating its sixtieth year of publication.^2^ However, historians, sociologists, and anthropologists of medicine and disease would rightly disagree, for the science of molecular biology did not develop until nearly three decades after the bacteriophages were first discovered in the early twentieth century. And in those intervening years between their discovery and deployment in molecular biology, the phages had already played key roles in multiple chapters of the history of medicine and public health. From the very time of their discovery, for instance, they were investigated as potential therapeutic agents against infectious bacterial diseases such as dysentery, cholera, and typhoid.^3^ Although this approach to infectious disease therapeutics underwent a long eclipse, especially in the West, the field of phage therapy is seeing an enormous revival now, a century later, both as a science and as a development worthy of sociological and anthropological scrutiny.^4^ Indeed, as noted by Charlotte Brives and Claas Kirchhelle, who in 2023 organised an interdisciplinary meeting on phages as ‘variable’ viruses, the variability of the phages is manifested not only biologically but also in multiple social contexts.^5^ In addition to what Brives, an anthropologist of science and medicine, previously described as the ‘pluribiotic’ character of phages,^6^ these viruses have shown variability in the contexts of the forms of knowledge developed in order to account for and understand their biological specificities, as well as the very diverse nature of projects for which they have been harnessed.^7^ In addition to their potential for therapy and as curious objects worthy of investigation in their own right, the phages were also very quickly adopted as tools in medical laboratories for a variety of purposes. The deployment of bacteriophages in areas of diagnostics and in the identification and differentiation of bacterial types for epidemiological purposes, similar to the way antigens and antibodies were used, has been well documented by various historians and STS scholars.^8^ This paper recounts a hitherto overlooked episode from the pre-molecular biology phase of the history of the bacteriophages, whereby they were used as yet another tool in medical laboratories.

Specifically, this article discusses the way in which bacteriophages became the vehicle for transferring the skills and know-how about fundamental laboratory techniques for working with viruses, even while there were lingering doubts about their own identity as viruses of bacteria.^9^ The primary driver for this transfer was the Australian medical researcher Frank Macfarlane Burnet (1899-1985), who, in the early 1930s, brought his dozen or so years of experience working with phages^10^ together with the then newly developed laboratory methods for virus cultivation in eggs^11^ to develop methods for growing and quantifying animal viruses. Thus deployed, the phages became the direct means of scaling up the production of influenza virus in the laboratory, a crucial process for the large-scale production of vaccines.^12^

Drawn primarily from the published literature, both scientific and personal, by central figures in this history, this paper offers a view from the ground, so to speak, of medical research in action during the first half of the twentieth century, when the discipline of virology was coming into its own. It highlights the way in which viruses during the interwar period came to occupy a space very similar to that described by the historian Andrew Mendelsohn, taken up by bacteria during the last decades of the nineteenth century.^13^ Just as he showed that the applied aspects of bacteriology in vaccine development during the 1880s and 1890s enabled bacteria to be ‘aligned […] with other living things’,^14^ I argue that similar practical concerns led to the treatment of viruses as living organisms in the laboratory, even as distinctions between them and living microbes such as bacteria were becoming clearer. The historian Ton van Helvoort’s suggestion that ‘bacteriological paradigm’^15^ shaped the study of viruses for decades is underscored by the work and words of the pioneers of virology as a discipline, including Burnet, who wrote in his memoirs:

The nature of the agent, whether virus in the modern sense or organism, puts no special imprint on the disease it causes. Malaria, due to a relatively large organism, is spread like the small arboviruses by mosquitoes… Anyone interested in the diseases they cause or how they are to be prevented must think of viruses as micro-organisms.^16^

Burnet’s description of the viruses and his practical use of the phages call up the notion of the ‘microbial turn’ in science, first described by anthropologists Heather Paxson and Stefan Helmreich,^17^ and more recently discussed and critiqued by Brives and Zimmer,^18^ as the shift in the understanding of microbes (including viruses) as integral members of the ecosystems they occupy rather than as mere pathogens. But rather than the advances in microbiology and microbial genomics that drove these scholars to call for a consideration of microbial communities as ‘model ecosystems’,^19^ I would argue that the microbial turn has its roots in the very origins of microbiology. After all, it was a comparison of the process of fermentation in wine that led the likes of Louis Pasteur and Joseph Lister to develop the so-called germ theory of disease in the late nineteenth century. And, more pertinently to the history at hand, in 1940, decades before the events highlighted by Paxson and Helmreich, Burnet had already emphasised the need for an ecological approach to studying and working with microbes, in a semi-popular book on the subject.^20^ Later, in his memoirs, he would make an even more direct declaration of precisely the notion of the microbial turn in biology: ‘Bacteria are enormously important in all sorts of inconspicuous roles, quite apart from their activity in causing disease.’^21^ Moreover, he explicitly linked this claim to the bacteriophages with the observation that ‘wherever bacteria are multiplying actively we are liable to find the viruses which can prey on them’.^22^

Burnet’s own introduction to, and understanding of, the phages was largely through the work and ideas of the microbiologist Félix d’Hérelle (1873-1949), who not only discovered these viruses and gave them their enduring label, but also introduced the techniques for their quantification and assay. The history of bacteriophage discovery, particularly the controversies surrounding the priority in discovery and early debates over its nature, has been extensively examined and discussed by both scientists and historians.^23^ But certain details of d’Hérelle’s early investigations, which have been overlooked or at least underplayed in most accounts, are of particular relevance to the historical episode detailed here. Therefore, I will reprise these details briefly before turning to Burnet’s story.

## Félix d’Hérelle: counting plaques to assay bacteriophages

D’Hérelle’s discovery of the bacteriophages emerged in the context of an outbreak of bacterial dysentery that occurred in the garrison town of Maisons-Laffitte, outside Paris. Although a small incident – the initial casualties included just ten infantrymen and two civilians domestics in late July and early August of 1915 – the symptoms were so unusually severe that Georges Bertillon, the physician in charge of the garrison, suspected that the cause was something other than the common dysentery-causing *Shigella* strains^24^ and assigned the investigation to d’Hérelle, a largely self-taught microbiologist with experience working on practical problems of infectious diseases of insects in Central and South America and North Africa. In short order, d’Hérelle found that the outbreak had indeed been caused by a hitherto unknown strain of *Shigella*, which he was then able to subject to a more detailed microbiological study throughout the course of the patients’ infection and convalescence. It was in the course of these studies that he discovered and named the bacteriophages.^25^

The results of d’Hérelle’s investigations were first presented by Emile Roux, then director of the Pasteur Institute, at the meeting of the French Academy of Sciences on 3 September 1917, and were published the following week.^26^ Whereas this initial report, out of necessity and tradition, had to be succinct, d’Hérelle later provided more details about his experiments and observations in his memoirs:

Several soldiers were treated at the hospital of Maisons Laffitte; I collected their stool samples for research. The bacteria that I had isolated had not yet been described, [although] the same germ had been found during the course of an epidemic in Scandinavia around the same time… I repeated the experiment which, with the grasshoppers had given me a result. I passed an emulsion of the dysentery stools in broth through a porcelain filter, mixed the filtrate with a culture of dysentery bacilli and placed the whole mixture in an incubator at 37°C; after a few hours of incubation I spread a drop of this mixture on a plate of nutrient agar and incubated it to look for the development of glassy plaques; I injected a few drops of the mixture into rabbits to see if they would contract dysentery. The rabbits remained perfectly healthy. When spread on agar, on two different occasions, glassy plaques dotted the surface of the dysentery bacilli cultures.^27^

D’Hérelle immediately drew parallels between the formation of the glassy plaques described in these passages – *taches vierges* in French – and the formation of colonies of bacteria when grown on solid media, and described the experiments using explicit bacteriological terminology to make the case that the phages ‘infected’ bacteria in much the same way as bacteria themselves did humans and animals:

If one adds to a culture of Shiga, a dilution of approximately one to a million of an already lysed culture, and… spreads out on an agar slant a drop of this culture, one obtains after incubation, a coat of dysentery bacilli showing a certain number of circles about 1 mm in diameter, where the culture is void; these points can only represent *the colonies of the antagonistic microbe.* ^28^

That the principles of serial dilution and plaque counting used by d’Hérelle were fundamentally sound is borne out by the fact that the methods for handling phages in the laboratory have remained basically unchanged more than a century later and form a part of the essential toolkit of every microbiologist and molecular biologist working with bacteriophages.

The contrast in the historical fate of the bacteriophages and d’Hérelle’s methods for working with them, versus his controversial ideas about the nature of the phages and their impact on his own career, is worth a brief mention here. In his as yet unpublished analysis of d’Hérelle’s career, philosopher and historian Emiliano Fruciano has pointed out that the early priority disputes regarding the discovery of the phenomenon of bacteriophagy (earlier dubbed as a transmissible lysis^29^) as well as the nature of the causative agent, played a big role in colouring the scientific community’s opinions about the trustworthiness of d’Hérelle’s claims and ideas.^30^ The Belgian microbiologist Jules Bordet (1870-1961) – who appears to have perceived in d’Hérelle’s claims about the nature of bacteriophage as a challenge to his own authority in immunology^31^ – was the first to cast doubt on d’Hérelle’s idea that bacteriophagy was caused by a virus. This battle, often combined with the issue of priority in discovery, was pursued by Bordet’s sometime associate André Gratia (1893-1950), for several more years, but there were other influential scientists, including later phage workers André Lwoff and Gunther Stent, who despite the lack of any concrete evidence, ‘*felt* that d’Hérelle may have been dishonest’ in his claims regarding his knowledge regarding Frederick Twort’s discovery of transmissible lysis.^32^ But, regardless of whether or not one bought into d’Hérelle’s ideas about the nature of the bacteriophage as an obligate parasite of bacteria, his experimental data were never called into question, and the practical laboratory methods that he had introduced for working with the phages were widely used in laboratories all over the world.^33^ Also true is that despite his many and influential detractors and his own volatile personality, d’Hérelle always had some support for his ideas, and was, in fact, nominated some 28 times for the Nobel prize between 1924 and 1937.^34^ Among those who were appreciative of d’Hérelle’s contributions towards phage research and somewhat sympathetic to his interpretations was Burnet, who, as mentioned earlier, obtained both his conceptual and technical introduction to the phages from the former’s publications.

## Macfarlane Burnet 1. Beckoned by bacteriophages

In his memoirs, which, unlike d’Hérelle’s *Autobiographie*, were published in his own lifetime, Burnet would recall that very soon after beginning his training as a resident in pathology at the Melbourne hospital in the early 1920s, a ‘curiously exciting book came into my hands’.^35^ This book was the English translation of *Le Bactériophage; Son Rôle dans l’Immunité*, d’Hérelle’s first book on the bacteriophages.^36^ Although Burnet could not recall how he encountered it, its contents evidently spurred his scientific imagination. The ‘fascinating new fairy-tale of science’,^37^ as he would later describe the bacteriophages, offered him opportunities to explore uncharted scientific territory, and he devoted the next dozen years or so to the study of different aspects of their properties and potential medical applications.^38^

One of Burnet’s earliest significant contributions to bacteriophage studies was to confirm the theoretical basis of d’Hérelle’s claim that counting plaques was a legitimate way to quantify phages, was to introduce some improvements to d’Hérelle’s assay.^39^ A big drawback in the earlier technique had been in the method for sampling the phages: d’Hérelle’s approach had been to grow the phages in a single test-tube of bacterial culture, and draw out small drop-sized samples that he would then check plaque formation at different dilutions. One problem with this method was that it treated the mixture as though the life cycles of all the phages in solution were synchronous, which is seldom or never the case in a population. Furthermore, d’Hérelle’s method also assumed a completely even distribution of phage particles, which again is statistically improbable.^40^ Burnet removed much of the randomness of d’Hérelle’s sampling technique by introducing some key modifications. Rather than follow phage growth in a single tube, he carried out the experiment in a series of capillary tubes, each containing the same volume of bacterial culture mixed with phage. Then, at fixed intervals over the course of several hours, he sampled ten of these capillary tubes at a time – each on one Petri dish – creating, in effect, multiple snapshots of the phage population at each point in time. He repeated this sort of sampling periodically over the course of several hours and so tracked the manner in which the phage particles increased over a period of time. He also repeated the set of experiments using a different pair of phage and host bacterium, and thus confirmed the fact that phage multiplication involved the same sequence of steps.^41^ In doing so, he not only improved the actual technique but also confirmed d’Hérelle’s notion that phage was indeed a virus, capable of replication only inside its bacterial host. His experiments also verified d’Herelle’s claims that phage multiplication proceeded in a stepwise fashion, with each rise in numbers representing a new round of phages being released into the medium from lysed bacteria.

Representing as they do, the first subject on which Burnet published a substantial body of research, the bacteriophages played a very significant role in his development as a biologist. In his very positive review of Burnet’s *Biological Aspects of Infectious Disease*, the renowned Cambridge University polymath J.B.S. Haldane (1892-1964) felt compelled to point out that, despite not being mentioned at all, it was Burnet’s work with phages that gave the book its intellectual heft and gravitas:

Dr. Burnet has not only studied diseases of men and guinea pigs but of bacteria also, though he doesn’t tell us so in this book… He has studied the bacteriophage, and perhaps this is what makes him so impartial between man and his microbic enemies.^42^

More immediately, the phages gave Burnet the hands-on expertise for working with viruses. His 1929 paper on growth – which was, in fact, his only publication on this particular aspect of the phage behaviour – provides a particularly good example of the role the phages played in shaping his experimental practice and quantitative thinking. Although phage research was Burnet’s main research pursuit from 1923 to 1937, he also began other investigations during this time, among them, the study of bacterial toxins produced by the genus *Staphylococcus* and the study of polioviruses. He had travelled to England in 1925 in order to work on his PhD, and upon its completion in 1927, had returned to Australia to take up a position at his old institute. Barely three years later, he was invited back to England on a two-year research fellowship at the National Institute for Medical Research (NIMR) in Hampstead Heath in London, specifically to work on animal viruses. And although he certainly made forays into research in the area of animal virology, he also continued with his phage work and maintained a steady output of publications on the topic throughout the fellowship period.^43^ During this time, he became acquainted with researchers in neighbouring laboratories, virologist Christopher Andrewes and physical chemist William Elford, who were conducting studies on estimating the sizes of viruses, including the bacteriophages, using techniques developed by the latter.^44^ Most of the phages they used for their estimations had been supplied by Burnet from his experiments, a fact that they acknowledged early in their paper.^45^ It was during this visit to England, thanks in part to his proximity to Andrewes’s laboratory and conversations about the work there, that Burnet first encountered and developed a long-term interest in influenza, about which he would later say, ‘From 1935 to 1955, one can summarize my life as learning about influenza viruses in chick embryos’.^46^

## Macfarlane Burnet 2. From bacteriophages to animal viruses

Having come of age during the First World War, Burnet had lived through the ‘Spanish flu’ pandemic – he was still a teenager when it began and a medical student when it ended. Indeed, he had even suffered ‘a relatively mild attack’ in August of 1919, which is when Australia suffered most seriously from the pandemic. Furthermore, he suffered a second attack of influenza in early 1932, along with two of his children, soon after the family had reached London for his NIMR fellowship.^47^ Consequently, he was well aware of the problems the disease presented, both to medical practitioners dealing with patients, as well as laboratory researchers who were trying to find the causative agent. As he put it, influenza ‘was *the* great challenge to the bacteriologists of the twenties’.^48^ The January 1932 London outbreak had been a rather ‘sharp’ one, Burnet recalled, and it has ‘stimulated the National Institute at Hampstead to make plans to investigate influenza when next it appeared’.^49^ They didn’t have to wait very long, for influenza appeared again at the end of that same year. When this happened, Christopher Andrewes together with his other virologist colleagues, Wilson Smith and Patrick Laidlaw, joined what must have been quite a competitive race among various laboratories worldwide to isolate and identify the causative agent, which by then was widely believed to be a virus rather than a bacterium. As Burnet recounted, what was required in order to isolate the putative virus:

…was to obtain infectious sputum or throat washings, filter the material through Elford membranes and inoculate the filtered fluid and any virus it contained into as many different species of animals as possible and by all the possible routes, by injection into veins or under the skin, blown up the nose and given by mouth.^50^

When Smith, Andrewes, and Laidlaw began their investigations, Burnet added, they followed this procedure, including among the animals to inoculate ferrets, which were a rather unusual model organism for medical studies, and very quickly thereafter, found the culprit.^51^

I fancy their thinking was that as the common laboratory animals had been tried in the past they should try the one unusual animal that was readily available to them. They had plenty of ferrets, Washings from the throats of influenza patients were dropped into ferret noses. The ferrets sneezed and ran a brief high temperature, It was ferret influenza: Laidlaw and company had holed out in one!^52^

Despite his keen interest in the findings of the influenza team, Burnet did not work on any aspect of this virus while at the NIMR, so as to avoid ‘poaching on someone’s preserve’.^53^ In fact, even though his NIMR fellowship had been awarded specifically to study viral diseases of humans or other animals, upon his arrival there in early 1932, he discovered that ‘all the viruses which were then available for study in relatively unspecialized laboratories, were under investigation by one or other members of the staff’.^54^ Luckily, he was soon assigned a problem by Sir Henry Dale (1875-1968) – the director of the NIMR, who was responsible for inviting Burnet in the first place – which unexpectedly gave rise to a long-term project. Dale himself was a physiologist (who would, in a few years, receive a Nobel Prize for his work on chemical transmission in nerve endings^55^), but had his fingers in many research pies, in part because of his position.^56^ One of these endeavours was a collaboration with some scientists in Germany to test the effectiveness of antimalarial drugs such as Atebrin. At that time, these drugs were being tested against a strain of bird malaria in canaries. Sometime in 1931, the canaries used in the tests became infected with a very virulent virus, and when Dale was asked for help in defining the nature of the pathogen, he assigned the problem to Burnet.^57^

The events of Burnet’s unfolding investigation of the canary viruses are reminiscent of the course of d’Herelle’s discovery of the bacteriophages. Both men resolved their assigned problems rather quickly; just as d’Herelle found a new strain of the dysentery bacillus, so too did Burnet quickly determine that the canary disease agent was ‘clearly a large virus related to fowlpox rather than, as the Germans had thought, a virus related to fowl plague’.^58^ Each then decided to conduct further studies on their discoveries, which, in both instances, were uncharted territory and hence, not only ripe for the picking but also fair game. At this point, the two men’s research trajectories changed. D’Hérelle, as described earlier, proceeded to track the infection in the patients, discover the bacteriophages, and devote the rest of his career to studying and utilising them. Burnet, on the other hand, used the canary pox virus as a tool to learn and expand on the knowledge about animal viruses more broadly. Interestingly, although this work did not materially involve them, the phages continued to play a part in Burnet’s scientific life, because the modifications that he introduced to the study of the canary pox virus were directly drawn from the lessons he had learned from his prior work.

## Macfarlane Burnet 3. Counting pocks to assay influenza viruses

The idea that developing chick eggs might be useful for the propagation of viruses had been considered as early as 1911 when pathologists Peyton Rous and James Murphy were able to demonstrate the growth of tumours on the chorioallantoic membranes of fertilised eggs when the eggs were injected with a cell-free filtrate of tumour tissue obtained from chicken sarcoma tumours.^59^ At the time, however, the viral identity of the tumour-inducing agent was still unknown, and consequently, the potential of the egg membranes for growing viruses was not capitalised upon. A couple of decades later, the American virologist Ernest Goodpasture at Vanderbilt University, who was looking for consistent and dependable techniques to study animal viruses in the laboratory with a view to develop vaccines against the pox viruses, chose fowl pox (of chickens) as his model system because the ‘infectious material is readily obtained and easily handled, since the disease is limited to fowls’.^60^ By then, many medical researchers had accepted Rous’s idea that chicken sarcomas were caused by viruses.^61^ Goodpasture, who was certainly in this camp, began to apply Rous and Murphy’s earlier observations about tumour transmissibility towards developing a technique for growing the fowl pox viruses in chick embryos.^62^

Burnet learned of Goodpasture’s work soon after his arrival in the UK and immediately thought to use it for his own canary pox studies, because ‘the fact that it produced a disease of *birds* immediately suggested that this would be a suitable virus to grow on the membrane of the chick embryo’.^63^ But he did not stop with merely growing the canary pox virus in developing eggs.^64^ Rather, he went several steps further, first to show the broad applicability of this experimental system to grow other animal viruses,^65^ and also – in a few years – to develop a reproducible technique for quantifying and assaying animal viruses.^66^ As he later explained:

The most important observation… was probably what I saw after a very weak solution of canary-pox virus had been placed on the membrane. The virus developed at only two points producing round opaque spots of proliferating cells a couple of millimetres across. Then I realized something I should have thought of months earlier, that by putting a series of consecutive dilutions, 1 in 10, 1 in 100, 1 in 1,000, etc., on to the egg membrane, at a certain dilution there would be a countable number of these spots or “pocks” and that this number multiplied by the dilution used would be a measure of the number of virus particles in the starting material… for several years this “pock counting” method on the chick embryo was the most useful way of measuring the activity of several viruses.^67^

The influence of phages in this technique is very much in evidence in the language that Burnet used to describe his approach to the technique, e.g. the serial dilutions and the emphasis on counting. Indeed, his entire line of reasoning bears more than a passing resemblance to the way d’Herelle described the dilution protocols for phages in his memoirs.^68^ Thus, although Burnet had stopped working actively on the phages after the 1930s (he published his last research paper mentioning the bacteriophages in 1937^69^), their legacy lived on through his work on the cultivation and assay of viruses in the developing egg.

Besides providing an easily assayable system, the chick embryo also proved useful in other ways for animal virus research. At NIMR, Burnet shared laboratory space with a veterinary scientist, Ian Galloway, who was studying diseases of livestock such as the foot and mouth disease and vesicular stomatitis. The success of the chick embryo system in cultivating the pox viruses had prompted the two men to also test the effects of Galloway’s cattle viruses in the same system. They found that although ‘superficially similar’, the viruses exerted very different effects on the chick embryo: whereas the foot and mouth virus didn’t even grow properly and had no visible effects, the vesicular stomatitis virus proliferated rapidly and killed the membrane tissues.^70^ Word evidently got around, and Burnet soon received other material to test, among them two more diseases afflicting chickens – Newcastle disease and a second disease at first erroneously believed to be a fowl plague.^71^ Much later, after he had begun to work on the influenza virus in Melbourne, Burnet would find out that the latter was, in fact, a strain of the influenza virus A.^72^ ‘But’, as he said, ‘it was still a long step before fowl plague turned out to be just a variety of influenza A. Quite a lot had first to be learnt about the behaviour of human influenza’.^73^

Burnet finally got his chance to work directly on the influenza problem very soon after returning to Melbourne upon the completion of his NIMR fellowship. In 1935, there was an epidemic outbreak of influenza, which occurred in his own backyard, so to speak, specifically at the Nurses’ Home of the Melbourne Hospital, and which he was called upon to investigate. ‘I had no difficulty in transmitting the infection to a ferret,’^74^ and from there, the isolation of the virus and its propagation in chick embryos were but short steps.^75^ Over the next two decades, influenza research would remain a predominant pursuit, despite his active engagement in other problems, resulting in a total output of ninety-eight papers on the subject between 1935 and 1958.^76^ A sizeable subset of these papers dealt with the cultivation of these viruses in the chick embryo membranes.

In addition to their susceptibility to a large variety of viruses, both Burnet and Goodpasture saw the potential of the fertilised eggs as a useful medium ‘for the production in quantity of uncontaminated vaccines’.^77^ For example, quite early in the course of his investigation of the Melbourne outbreak, Burnet predicted that:

In the near future it is highly probable that attempts at immunization of human beings with killed or attenuated influenza virus will be made, and here the egg membrane technique may find a very important practical application. It allows growth of influenza virus which is certainly free from contaminating viruses or bacteria and *a priori* should almost certainly provide a much more suitable “raw material” for the preparation of antigens than either ferret or mouse tissues.^78^

Sure enough, Burnet’s predictions came to pass quite soon and the development of successful vaccines against influenza did indeed owe much to the fact that their production could be scaled up quite easily in the humble egg.^79^

The intellectual debt that these vaccines also owe to the bacteriophages is perhaps less direct, but it is a debt that Burnet himself acknowledged, even if only obliquely, in various writings and speeches in later life. Perhaps the most explicit expression of the connection was his labelling of a lecture for a 1958 symposium on immunity and virus infections as ‘From bacteriophage to influenza virus,’ in which he devoted the lion’s share of the talk to the former.^80^ He claimed, admittedly with some oversimplification, that: ‘My switch between 1935 and 1937 from phage to influenza virus was primarily because the Melbourne strain of influenza A could be adapted to produce foci on the chorioallantois.’^81^ He also pointed out that, ‘Studies on influenza virus have paralleled in many ways what has been done with bacterial viruses. With chick embryos for cultivation and hemagglutinin tests for titrations, a well adapted strain is almost as easy to handle in the laboratory as a bacterial virus.’^82^

The parallels Burnet observed were, in some ways, reflected in the fates of the phages and the chick embryo techniques in his own laboratory, both coming to an end there due to the march of scientific progress as it were. The publication of the now classic paper by Emory Ellis and Max Delbrück on the growth of bacteriophages in 1939^83^ – a paper that cited Burnet’s 1929 paper on growth but puzzlingly skimmed over its implications for the nature of phages as viruses^84^ – marked a decisive turn in phage research from medical problems to molecular biology.^85^ Later in his memoirs, Burnet would confess to being ‘positively schizophrenic’ about molecular biology, and ‘found myself resenting the arrogance which defines biology as the chemistry of the nucleic acids, thus eliminating those aspects of the study of life and of its appreciation which have come my way’.^86^ That attitude and the fact that he had already moved on to working on animal viruses by this time, meant that neither he nor any of the students in his laboratory ever returned to active work on bacteriophages. Meanwhile, Burnet lamented, progress of a different sort caused the chick embryo technique for animal virus assays to be ‘displaced probably forever by the tissue culture techniques’, not only in his own lab but in research labs all over the world.^87^

## Conclusion

Even as tissue culture techniques made the chick-embryo–based techniques for the cultivation and assay of viruses obsolete, they became the medium for a similar application of the assay techniques of the bacteriophages towards quantifying animal viruses. This transfer took place some decades later in a laboratory far removed both geographically and in its scientific aims from Burnet’s lab in Melbourne. But the episode is worth a brief mention because it underscores the key part played by bacteriophage expertise and research for work on animal viruses. During the 1950s, Renato Dulbecco (1914-2012) – the first scientist who developed successful plaque-counting assay techniques for animal viruses on tissue culture^88^ – explicitly switched his research focus from the bacteriophages to animal viruses with the aim of applying the lessons learned from the former to develop the latter.^89^ The history of Dulbecco’s scientific career – from his beginnings as a physicist in Italy, to training with the American Phage Group, the switch to animal virology, and eventual specialisation in tumour virology, which last garnered him a share in the 1975 Nobel Prize in Physiology or Medicine^90^ – have been written about in considerable detail elsewhere,^91^ and I will not dwell on the subject here. But I will offer the following comment Dulbecco made to his oral history interviewer by way of support for my contention that the phages were the training ground for teaching future animal virologists: ‘My goal was to develop a plaque system like that of phage, because that is the way you can get a good assay.’^92^ Dulbecco also emphasised the importance of the assay in his contribution to his former phage mentor, Max Delbrück’s *festschrift*, with the title, ‘The plaque technique and the development of *quantitative* animal virology’ (emphasis added).^93^

Dulbecco’s title also brings to the fore one of the main broader historical points that I wished to highlight in the paper, namely, the importance of quantification and measurement, especially in the early stages of the investigation of something new, not only in the medical sciences, as shown here, but in science in general. The importance of this quantitative aspect of doing science is particularly exemplified in the uneven, stuttered progress of different branches of virology.^94^ For instance, although the animal viruses were among the earliest viruses to be discovered in the late nineteenth century, decades well before the first bacteriophages,^95^ research on those viruses only progressed in fits and starts well into the early decades of the twentieth century. As late as 1931, for instance, the American pathologist Earl McKinley would make the case for the importance of methods for culturing and quantification for virology to advance further:

Progress has been slow and no doubt will continue to be slow until some of the fundamentals are established… The cultivation of the first bacterium on artificial medium gave to the science of bacteriology a fundamental working tool. Looking ahead in the field of the filterable viruses our greatest need is for working tools, new ideas and methods of approach.^96^

As I have shown in this paper, animal virology only advanced when methods for growing viruses and counting first pocks first on chick embryos and then plaques on cultured cells *in vitro*, were developed. As cancer virologist Harry Rubin remarked in his own contribution to the Delbrück *festschrift*, which, like Dulbecco’s essay, emphasised quantification in its very title, ‘it is safe to say… that there was little increase in our understanding of how viruses cause cancer until quantitative methods were introduced for studying virus-cell interactions’.^97^ Rubin, it should be noted, had worked as a post-doc in Dulbecco’s lab, where together with Howard Temin, a PhD student in the laboratory, he had developed a cancer virus assay analogous to the plaque and pock counting methods of the phages and infectious animal viruses. Rather than killing cells and forming plaques, the cancer viruses transformed the cells they infected and so produced a different sort of countable effect in tissue culture.^98^

In contrast to the animal viruses, research on the bacteriophages took off within a very few years of d’Hérelle’s 1917 discovery.^99^ It proceeded at a fairly steady pace with an output of about 120 publications per year between 1922 and 1941, as evidenced by a bibliography of publications on phages compiled by a German microbiologist, Hansjürgen Raettig.^100^ The exponential rise in phage publications after the Second World War, also shown in Raettig’s volume, may be attributed to the deployment of bacteriophages as a tool in molecular biology. But the reason for the initial steady progress of phage research was, I contend, that the discovery of the phages was based on the very same property – inducing bacterial lysis – that caused them to form the plaques, which in turn, was the basis for their quantification and ability to be assayed. Hand-in-glove with this quantification came such other attributes to animal virology, such as applicability – e.g. towards scaling up the influenza vaccines – experimental rigour, and a measure of respectability, which is what scientists like Burnet and Dulbecco brought to the table for the science of animal viruses when they carried over the techniques of counting plaques from their studies on bacteriophages.

The story of the application of the techniques of bacteriophage research in other areas of medical virology has perhaps suffered a fate that is all too common in history, namely, being overlooked simply because of their matter-of-fact, almost mundane, quality. The episodes recounted in this paper do not share the common attributes of historically memorable events, either in or out of science, being neither controversial nor obviously startling or earth-shaking in their outcomes. And yet, or maybe even because of that everyday quality, these episodes have important takeaways for historians and scientists alike. More than the exciting Eureka moments or grand debates that are remembered, the work of the type conducted by Burnet and Dulbecco is important and instructive because it forms the bricks and mortar of everyday science. The ways in which techniques honed on bacterial viruses proved applicable in working with animal viruses have implications of continuing relevance to this day, for virology in particular, but also for biomedical research and even medical practice more generally. They also illustrate the role and value of maintaining a broad vision and flexible thinking as well as the dangers of tunnel vision and compartmentalising knowledge. There was no way either Burnet or Dulbecco could have guessed, when they embarked on and pursued problems concerning the bacteriophages, that the habits of mind, discipline, and techniques they learned from the experience would help them in the years to come in addressing problems in the areas of influenza or cancer. And yet that is what happened.

## Footnotes

1

Nicholas C. Mullins , ‘The Development of a Scientific Specialty: The Phage Group and the Origins of Molecular Biology’, Minerva, 10, 1 (1972), 51–82 ; Pnina G. Abir-Am , ‘Essay Review: How Scientists View Their Heroes: Some Remarks on the Mechanism of Myth Construction’, Journal of the History of Biology, 15, 2 (1982), 281–315 11611044 10.1007/BF00233019; William C. Summers , ‘How Bacteriophage Came To Be Used by the Phage Group’, Journal of the History of Biology, 26, 2 (1993), 255–667 11623160 10.1007/BF01061969; Angela N.H. Creager , ‘The Paradox of the Phage Group: *Essay Review’* , Journal of the History of Biology, 43, 1 (2010), 183–9320665234 10.1007/s10739-010-9226-8.

2 John Cairns, G.S. Stent, and J.D. Watson (eds), *Phage and the Origins of Molecular Biology* (Cold Springs Harbor, NY: Cold Spring Harbor Laboratory of Quantitative Biology, 1966).

3 Felix d’Hérelle, *Le bactériophage: son rôle dans l’immunité* [The bacteriophage: its role in immunity] (Paris: Masson et cie, 1921); F. d’Hérelle , ‘An Address on Bacteriophagy and Recovery from Infectious Diseases’, Canadian Medical Association Journal, 24, 5 (1931), 619–2820318284 PMC382443, http://www.ncbi.nlm.nih.gov/pmc/articles/PMC382443/; F. d’Hérelle, ‘Bacteriophage as a Treatment in Acute Medical and Surgical Infections’, *Bulletin of the New York Academy of Medicine*, 7, 5 (1931), 329–48, http://www.ncbi.nlm.nih.gov/pubmed/19311785.

4

R. M Carlton , ‘Phage Therapy: Past History and Future Prospects’, Archivum Immunologiae et Therapiae Experimentalis, 47, 5 (1999), 267–7410604231 ; Emiliano Fruciano and Shawna Bourne , ‘Phage as an Antimicrobial Agent: D’Hérelle’s Heretical Theories and Their Role in the Decline of Phage Prophylaxis in the West’, The Canadian Journal of Infectious Diseases & Medical Microbiology, 18, 1 (2007), 19–26, http://europepmc.org/articles/pmc2542891, accessed 2 February 201518923687 10.1155/2007/976850PMC2542891; A. Dublanchet and E. Fruciano , ‘Brève histoire de la phagothérapie’, Médecine et Maladies Infectieuses, 38, 8 (2008), 415–20, https://www.sciencedirect.com/science/article/pii/S0399077X0800173X 18692974 10.1016/j.medmal.2008.06.016; Nina Chanishvili , ‘Phage Therapy – History from Twort and d’Hérelle through Soviet Experience to Current Approaches’, Advances in Virus Research, 83 (2012), 3–40 22748807 10.1016/B978-0-12-394438-2.00001-3; William C. Summers , ‘The Strange History of Phage Therapy’, Bacteriophage, 2, 2 (2012), 130–323050223 10.4161/bact.20757PMC3442826; Dmitriy Myelnikov , ‘An Alternative Cure: The Adoption and Survival of Bacteriophage Therapy in the USSR, 1922–1955’, Journal of the History of Medicine and Allied Sciences, 73, 4 (2018), 385–411 30312428 10.1093/jhmas/jry024PMC6203130; *idem* ‘Creature Features: The Lively Narratives of Bacteriophages in Soviet Biology and Medicine’, *Notes and Records: The Royal Society Journal of the History of Science*, 74, 4 (2020), 579–97; Charlotte Brives and Thomas Bonnin, ‘Phage Therapy Now and Then: Continuing Epistemic Issues in the Development of a “Misfit” Therapy’ (2019), https://halshs.archives-ouvertes.fr/halshs-02486744.

5

Charlotte Brives and Claas Kirchhelle , ‘Introduction to the Special Issue: Variable Viruses – The Past, Present, and Future of Bacteriophage Research’, PHAGE: Therapy, Applications, and Research, 5, 1 (Special Issue: Variable Viruses) (March 2024), 1–3, https://www.liebertpub.com/doi/10.1089/phage.2024.0007, accessed 21 March 2024.

6 Charlotte Brives, *Face à l’antibiorésistance. Une Écologie Politique Des Microbes* (Amsterdam editions, 2022), https://journals.openedition.org/lectures/58048, accessed 19 May 2025.

7 Brives and Kirchhelle, *op. cit.* (note 5).

8

Claas Kirchhelle , ‘The Forgotten Typers: The Rise and Fall of Weimar Bacteriophage-Typing (1921–1935)’, Notes and Records: The Royal Society Journal of the History of Science, 74, 4 (2020), 539–65; Claas Kirchhelle et al., ‘Reuse, Repurpose, and Recycle: Bacteriophages and Microbial Surveillance (1921–2023)’, *PHAGE: Therapy, Applications, and Research*, 5, 1 (Special Issue: Variable Viruses) (2024), 14–21; Claas Kirchhelle and Charlotte Kirchhelle, ‘Northern Normal: Laboratory Networks, Microbial Culture Collections, and Taxonomies of Power (1939–2000)’, *Engaging Science, Technology, and Society*, 10, 1–2 (2024), https://estsjournal.org/index.php/ests/article/view/1445, accessed 15 June 2025.

9 For details about the views of different sides of those embroiled in the debate, see F. d’Hérelle et al., ‘Discussion on the Bacteriophage (Bacteriolysin)’, The British Medical Journal, 2, 3216 (1922), 289–99. For historical accounts of such debates, see also Alan W. Varley, ‘Living Molecules or Autocatalytic Enzymes: The Controversy Over the Nature of Bacteriophage 1915–1925’ (unpublished PhD thesis: University of Kansas, History, 1986); Ton van Helvoort , ‘Bacteriological and Physiological Research Styles in the Early Controversy on the Nature of the Bacteriophage Phenomenon’, Medical History, 36, 3 (1992), 243–701518340 10.1017/s0025727300055265PMC1036587; W.C. Summers , Felix d’Herelle and the Origins of Molecular Biology (New Haven, CT: Yale University Press, 1999), 1–46 .

10 A.W. Varley, ‘Early Bacteriophage Research: The Contribution of Frank Macfarlane Burnet’(MSc: University of Kansas, 1981); Neeraja Sankaran, ‘Frank Macfarlane Burnet and the Nature of the Bacteriophage, 1924–1937’ (unpublished PhD thesis: Yale University, 2006).

11

Alice Miles Woodruff and Ernest W. Goodpasture , ‘The Susceptibility of the Chorio-Allantoic Membrane of Chick Embryos to Infection with the Fowl-Pox Virus’, The American Journal of Pathology, 7, 3 (1931), 209–222 19969963 PMC2062632.

12 E.L. French, ‘Burnet and the Biology of Animal Viruses’, *Walter and Eliza Hall Institute of Medical Research Annual Review: A Tribute to Sir Macfarlane Burnet* (1978), 14–9.

13

J.A. Mendelsohn , ‘“Like All That Lives”: Biology, Medicine and Bacteria in the Age of Pasteur and Koch’, History and Philosophy of the Life Sciences, 24, 1 (2002), 3–36 12664951 10.1080/03919710210001714293.

14
*Ibid.*, 3.

15

T. van Helvoort , ‘A Bacteriological Paradigm in Influenza Research in the First Half of the Twentieth Century’, History and Philosophy of the Life Sciences, 15, 1 (1993), 3–21 8310118 ; Also by the same author, ‘A comment on the early influenza virus vaccines: the role of the concept of virus’, in Stanley A. Plotkin and Bernadino Fantini (eds), *Vaccinia Vaccination Vaccinology: Jenner, Pasteur and Their Successors* (Paris: Elsevier, 1996), 193–7.

16 F.M. Burnet, *Changing Patterns: An Atypical Autobiography* (Melbourne: Heinemann, 1968), 93. See also Thomas M. Rivers , ‘Viruses and Koch’s Postulates’, Journal of Bacteriology, 33, 1 (1937), 1–12 16559982 10.1128/jb.33.1.1-12.1937PMC545348.

17

Heather Paxson and Stefan Helmreich , ‘The Perils and Promises of Microbial Abundance: Novel Natures and Model Ecosystems, from Artisanal Cheese to Alien Seas’, Social Studies of Science, 44, 2 (2014), 165–93, https://journals.sagepub.com/doi/10.1177/0306312713505003, accessed 15 June 202524941610 10.1177/0306312713505003.

18 Charlotte Brives and Alexis Zimmer, ‘Écologies et Promesses Du Tournant Microbien’ [Ecologies and promises of the microbial turn], *Revue d’anthropologie Des Connaissances*, 15, 15–3 (2021), https://journals.openedition.org/rac/24895, accessed 19 May 2025.

19 Paxson and Helmreich *op. cit.* (note 17).

20 F. Macfarlane Burnet, *Biological Aspects of Infectious Disease*, 1st edn (Cambridge: Cambridge University Press, 1940). The popularity and influence of this book are evident from the fact that two further updated editions of the book were published during his own lifetime in 1953 and 1962 under a slightly different title: *The Natural History of Infectious Disease.*

21 Burnet, *op. cit.* (note 16), 58.

22
*Ibid.*

23 See, for example, Donna H. Duckworth , ‘Who Discovered Bacteriophage?’ Bacteriological Reviews, 40, 4 (1976), 793–802 795414 10.1128/br.40.4.793-802.1976PMC413985; Antony Twort, *In Focus, out of Step: A Biography of Frederick William Twort FRS, 1877-1950* (Stroud, UK: Alan Sutton Publishing Ltd, 1993); Varley, *op. cit.* (note 9); Summers, *op. cit.* (note 9), 47–96.

24

Georges Bertillon , ‘Une Épidémie de Dysenterie Hémorragique Dans Un Escadron de Dragos’ [An epidemic of unspecialised dysentery in a squadron of dragoons], Annales de l’Institut Pasteur, 30, 3 (1916), 141–44.

25 Although the common interpretation of bacteriophage is as ‘bacteria-eaters’ from the Greek root ‘phago’ meaning to eat, d’Hérelle would emphasise the fact that he intended the name to be used in the scientific sense of ‘developing at the expense of’ (Félix in d’Hérelle, *Le Bactériophage; Son Rôle Dans l’immunité* [Masson et cie, 1921]; Félix d’Hérelle, *The Bacteriophage: Its Role in Immunity*, George H. Smith [trans.] [Baltimore: Waverly Press, 1922], 21).

26

Félix d’Hérelle , ‘Sur Un Microbe Invisible Antagoniste Des Bacilles Dysentériques’, Comptes Rendus de l’Académie Des Sciences, 145 (1917), 373–5 (For longer quotations from this paper, I have used the translation by Summers, *op. cit.* (note 9), 185–6, but cited page numbers of the original publication in French).

27 Félix d’Hérelle, *Autobiographie de Félix d’Hérelle: Les Pérégrinations d’un Bacteriologiste*, Alain Dublanchet (ed.) (Paris: Editions Médicales Internationales, 2017), 135. According to Summers *op. cit.* (note 9), 192, d’Hérelle composed his memoirs or *Autobiographie* between 1939 and 1946, but did not publish them during his lifetime. Copies of two versions appear to have survived and left in the possession of his grandson, Claude Hubert Mazure-d’Hérelle, who shared one with the Pasteur Institute in Paris, and the other – a later and more complete version – with the French microbiologist, Alain Dublanchet, who published it in the centenary year of d’Hérelle’s discovery. Passages appearing in this paper are my translations from Dublanchet’s edited publication.

28 D’Hérelle, *op. cit.* (note 26), 374, emphasis added.

29 See, for example, Frederick W. Twort , ‘An Investigation on the Nature of Ultra-Microscopic Viruses’, The Lancet, 186, 4814 (1915), 1241–3.

30 Emiliano Fruciano, ‘*The Impact of Human Interactions on the First Decades of Phage Research: The Complicated Story of Félix d’Herelle and His Studies at the Pasteur Institute (1917–1949)*’ (n.d.).

31

William C. Summers , ‘The Discovery of Bacteriophages and the Historical Context’, in David R. Harper et al. (eds), Bacteriophages: Biology, Technology, Therapy (Cham: Springer International Publishing, 2017), 1–15 , 8, 387–400.

32 Duckworth *op. cit.* (note 23), 793; Fruciano *op. cit.* (note 30), 6.

33 See F.M. Burnet , ‘A Method for the Study of Bacteriophage Multiplication in Broth’, British Journal of Experimental Pathology, 10, 2 (1929), 109–15, (109–10) for an assessment of the then extant interpretations of the phenomenon of bacteriophagy.

34 See Summers 1998 *op. cit.* (note 9); Fruciano n.d. *op. cit.* (note 30); and https://www.nobelprize.org/nomination/archive/show_people.php?id=2649.

35 F.M. Burnet, *op. cit.* (note 16), 51.

36 D’Hérelle, *op. cit.* (note 25).

37 Burnet, *op. cit.* (note 16), 52.

38 See *ibid.*, 51–62, for Burnet’s own reprisal of his achievements in this area.

39 Burnet, *op. cit.* (note 31).

40 See Emory L. Ellis and Max Delbrück , ‘The Growth of Bacteriophage’, The Journal of General Physiology, 22, 3 (1939), 365–8419873108 10.1085/jgp.22.3.365PMC2141994, for a detailed analysis of how d’Hérelle’s basic technique was improved upon. See also Emory L. Ellis, ‘Bacteriophage: One-Step Growth’, in J. Cairns et al. (eds), *Phage and the Origins of Molecular Biology* (Cold Springs Harbor, NY: Cold Spring Harbor Laboratory of Quantitative Biology, 1966), 53–62. Varley, *op. cit.* (note 10); Sankaran, *op. cit.* (note 10); and Neeraja Sankaran , ‘Stepping-Stones to One-Step Growth: Frank Macfarlane Burnet’s Role in Elucidating the Viral Nature of the Bacteriophages’, Historical Records of Australian Science, 19, 1 (2008), 91–92 .

41 Burnet, *op. cit.* (note 31), 112–3.

42 J.B.S. Haldane, ‘Pity the Poor Measle’, *World Review*, 1941, 57–9.

43 Burnet, *op. cit.* (note 16), 62.

44

W.J. Elford , ‘A New Series of Graded Collodion Membranes Suitable for General Bacteriological Use, Especially in Filterable Virus Studies’, Journal of Pathology and Bacteriology, 34 (1931), 505–21; W.J. Elford and C.H. Andrewes , ‘The Sizes of Different Bacteriophages’, British Journal of Experimental Pathology, 13, 5 (1932), 446–56; ‘Estimation of the Size of a Fowl Tumour Virus by Filtration through Graded Membranes’, *British Journal of Experimental Pathology*, 16, 1 (1935), 61–66; W.J. Elford , ‘Centrifugation Studies: I. Critical Examination of a New Method as Applied to the Sedimentation of Bacteria, Bacteriophages and Proteins’, British Journal of Experimental Pathology, 17, 5 (1936), 399–422 ; W.J. Elford and Christopher H. Andrewes, ‘Centrifugation Studies: II. The Viruses of Vaccinia, Influenza and Rous Sarcoma’, *British Journal of Experimental Pathology*, 17, 5 (1936), 422–30.

45 Elford and Andrewes, ‘Sizes of different bacteriophages’, *ibid.*, 447.

46 Burnet, *op. cit.* (note 16), 125.

47
*Ibid.*, 121.

48
*Ibid.*, 122, emphasis in original.

49
*Ibid.*

50
*Ibid.*

51

Wilson Smith, C.H. Andrewes , and P.P. Laidlaw , ‘A Virus Obtained from Influenza Patients’, The Lancet, 222, 5732 (1933), 66–68 .

52 Burnet, *op. cit.* (note 16), 122–23. A fuller account of how these investigators had ferrets in their possession is provided in these pages and also *idem.*, 48–49.

53
*Ibid.*, 89.

54
*Ibid.*

55 NobelPrize.org, ‘The Nobel Prize in Physiology or Medicine 1936’, https://www.nobelprize.org/prizes/medicine/1936/summary/.

56 NobelPrize.org, ‘Sir Henry Dale – Facts’, https://www.nobelprize.org/prizes/medicine/1936/dale/biographical/.

57 Burnet, *op. cit.* (note 16), 89–90.

58
*Ibid*; The results of this study conducted in collaboration with a colleague at NIMR were published a couple of years later: F.M. Burnet and J.E. Barnard , ‘A Virus Disease of the Canary of the Fowl-Pox Group’, The Journal of Pathology and Bacteriology, 37, 1 (1933), 107–22.

59

Peyton Rous and James B. Murphy , ‘Tumor Implantations in the Developing Embryo: Experiments with a Transmissible Sarcoma of the Fowl’, Journal of the American Medical Association, 56, 10 (1911), 741–42.

60 Woodruff and Goodpasture, *op. cit.* (note 11), 209.

61 For a detail account of the reception to Rous’s discovery, see Eva Becsei-Kilborn , ‘Scientific Discovery and Scientific Reputation: The Reception of Peyton Rous’ Discovery of the Chicken Sarcoma Virus’, Journal of the History of Biology, 43, 1 (2010), 111–5720503720 10.1007/s10739-008-9171-y; Neeraja Sankaran, *A Tale of Two Viruses: Parallels in the Research Trajectories of Tumor and Bacterial Viruses* (Pittsburgh, PA: University of Pittsburgh Press, 2021), 34–44.

62 Woodruff and Goodpasture, *op. cit.* (note 11). See also Ernest W. Goodpasture , ‘Some Uses of the Chick Embryo for the Study of Infection and Immunity’, American Journal of Epidemiology, 28, 1 (1938). 111–29; ‘Virus Infection of the Chick Embryo’, *Annals of Internal Medicine*, 13, 1 (1939), 1–11; ‘The Developing Egg as a Culture Medium’, *Journal of Laboratory and Clinical Medicine*, 26 (1940), 242–49.

63 Burnet, *op. cit.* (note 16), 90, emphasis in original.

64 Burnet and Barnard, *op. cit.* (note 58).

65

F.M. Burnet , ‘The Propagation of the Virus of Infectious Laryngo-Tracheitis on the Chorio-Allantoic Membrane of the Developing Egg’, British Journal of Experimental Pathology, 15, 1 (1934), 52–55 ; F.M. Burnet and I.A. Galloway , ‘The Propagation of the Virus of Vesicular Stomatitis in the Chorio-Allantoic Membrane of the Developing Hen’s Egg’, British Journal of Experimental Pathology, 15, 2 (1934), 105–13.

66 Frank Macfarlane Burnet, *The Use of the Developing Egg in Virus Research *(** Medical Research Council Special Reports 220. Medical Research Council, 1936).

67 Burnet, *op. cit.* (note 16), 91.

68 See d’Hérelle, *op. cit.* (note 26), 141.

69

F. Macfarlane Burnet and M. Freeman , ‘A Comparative Study of the Inactivation of a Bacteriophage by Immune Serum and by Bacterial Polysaccharide’, Australian Journal of Experimental Biology and Medical Science 15 (1937), 49–61 .

70 Burnet and Galloway, *op. cit.* (note 65).

71

F.M. Burnet and J.D. Ferry , ‘The Differentiation of the Viruses of Fowl Plague and Newcastle Disease: Experiments Using the Technique of Chorio-Allantoic Membrane Inoculation of the Developing Egg’, British Journal of Experimental Pathology, 15, 1 (1934), 56–64 .

72 Burnet, *op. cit.* (note 16), 129.

73
*Ibid.*, 125.

74
*Ibid.*

75

F.M. Burnet , ‘Influenza Virus Isolated from an Australian Epidemic’, Medical Journal of Australia 2 (1935), 651–53; ‘Propagation of the Virus of Epidemic Influenza on the Developing Egg’, *Medical Journal of Australia*, 2 (1935), 687–9.

76 Burnet, *op. cit.* (note 16), 4–5.

77 Burnet *op. cit.* (note 66); Goodpasture, ‘Developing Egg as a Culture Medium’, *op. cit.* (note 60), 242.

78 Burnet, ‘Propagation of virus of epidemic influenza’, *op. cit.* (note 65), 689.

79

F.M. Burnet , ‘The Influenza Virus’, Scientific American, 188, 4, 1953, 27–31 .

80 F.M. Burnet, ‘From Bacteriophage to Influenza Virus’, in V.A. Najjar (ed.), *Immunity and Virus Infection* (New York: Wiley, 1959), 164.

81
*Ibid.*, 172.

82
*Ibid.*, 173.

83 Ellis and Delbrück, ‘The Growth of Bacteriophage’, *op. cit.* (note 40).

84 See Sankaran, ‘Stepping-stones’, *op. cit.* (note 40), 92-93 for a detailed analysis of this point.

85 Sankaran, ‘Burnet and nature of bacteriophage’, *op. cit.* (note 40), 257–58.

86 Burnet, *op. cit.* (note 16), 175.

87

F.M. Burnet , ‘The Influence of a Great Pathologist: A Tribute to Ernest Goodpasture’, Perspectives in Biology and Medicine, 16, 3 (1973), 339 10.1353/pbm.1973.00304574439.

88

Renato Dulbecco , ‘Production of plaques in monolayer tissue cultures by single particles of an animal virus’, Proceedings of the National Academy of Sciences, 38, 8 (1952), 747–5210.1073/pnas.38.8.747PMC106364516589172; Renato Dulbecco and Marguerite Vogt, ‘Some problems of animal virology as studied by the plaque technique’, *Cold Spring Harbor Symposia on Quantitative Biology*, 18 (1953), 273–79.

89 Renato Dulbecco, Interview by Shirley K. Cohen, September 9 and 10, 1998. Caltech Oral Histories, http://oralhistories.library.caltech.edu/26/1/OH_Dulbecco_R.pdf.

90 NobelPrize.org, ‘The Nobel Prize in Physiology or Medicine 1975’, https://www.nobelprize.org/prizes/medicine/1975/summary/.

91

Daniel J. Kevles , ‘Renato Dulbecco and the New Animal Virology: Medicine, Methods, and Molecules’, Journal of the History of Biology, 26, 3 (1 October 1993), 409–4211613167 10.1007/BF01062056; David Baltimore, ‘Renato Dulbecco’, *Biographical Memoirs of the National Academy of Sciences*, 2014, 1–13; Sankaran, *Tale of Two Viruses*, *op. cit.* (note 61), 173–6.

92 Dulbecco, *op. cit.* (note 87), 26.

93 Renato Dulbecco, ‘The plaque technique and the development of quantitative animal virology’, in J. Cairns, G.S. Stent, and J.D. Watson (eds), *Phage and the Origins of Molecular Biology* (Cold Spring Harbor Laboratory of Quantitative Biology, 1966), 287–91 (emphasis added).

94 For an overview of the progress of animal virology, see the now classic review, Daniel J. Kevles and Gerald L. Geison , ‘The experimental life sciences in the twentieth century’, Osiris, 2nd Series, 10, (1 January 1995), 108–1810.1086/36874511618355. While I have not discussed the history of plant virology in this paper, the progress of which proceeded at a pace intermediate between the phages and animal viruses, it is not because I don’t recognise its importance, but because it proceeded quite independently, with little or no direct or explicit influence from phage research. (See H. Fraenkel-Conrat , ‘Portraits of Viruses: Tobacco Mosaic Virus’, Intervirology, 15, 4 [1981], 177–897028678 10.1159/000149230; Karen-Beth G. Scholthof et al., ‘Tobacco Mosaic Virus: One Hundred Years of Contributions to Virology’, Journal of the History of Biology, 33, 3 [2000], 604–6 for details about the progress of this branch of virology.)

95

Friedrich Löffler , and Paul Frosch , ‘Berichte Der Kommission Zur Erforschung Der Maul-Und Klauenseuche Bei Dem Institut Für Infektionskrankheiten in Berlin’ [Reports of the commission for research into foot-and-mouth disease at the Institute for Infectious Diseases in Berlin], Zentralblatt fur Bakteriologie, Parasitenkunde und Infektionskrankheiten, 28 (1898), 371–91.

96

Earl B McKinley , ‘The Filterable Viruses’, The Scientific Monthly, 32, 5 (1931), 398–403 .

97 Harry Rubin, ‘Quantitative Tumor Virology’, in J. Cairns, G.S. Stent, and J.D. Watson (eds), *Phage and the Origins of Molecular Biology* (Cold Spring Harbor Laboratory of Quantitative Biology, 1966), 292.

98 Howard M. Temin and Harry Rubin, ‘Characteristics of an Assay for Rous Sarcoma Virus and Rous Sarcoma Cells in Tissue Culture’, *Virology*, 6, 3 (1958), 669–88. Having come to Dulbecco’s laboratory as a veterinary scientist with little or no exposure to the bacteriophages, Rubin’s role in harnessing the latter to advancing animal virology was indirect at best and hence, his own story is best told elsewhere.

99 Duckworth *op. cit.* (note 23), 797.

100 Hansjürgen Raettig, *Bakteriophagie, 1917 Bis 1956: Zugleich Ein Vorschlag Zur Dokumentation Wissenschaftlicher Literatur* (Stuttgart: G. Fischer, 1958). The exponential rise in phage publications after the Second World War may be attributed to their deployment as a tool in molecular biology, a fact that is also borne out in the bibliography.

